# Nutrition education and its public health impact in Saudi Arabia: a systematic review

**DOI:** 10.3389/fpubh.2025.1700254

**Published:** 2025-11-19

**Authors:** Sarah N. Alsharif

**Affiliations:** Department of Clinical Nutrition, Faculty of Applied Medical Sciences, King Abdulaziz University, Jeddah, Saudi Arabia

**Keywords:** impact, nutrition, education, interventions, review, Saudi

## Abstract

**Background:**

Inadequate nutritional awareness may lead to harmful eating habits and poor diet quality. Nutrition education interventions have been shown to improve nutritional knowledge and behaviors.

**Aim:**

To assess the impact of nutrition education in Saudi Arabia, I reviewed relevant studies published between 2017 and 2024.

**Methods:**

For the present systematic review, PubMed, Scopus, Google Scholar, Web of Science, and the Cochrane Library were searched. A total of 12 relevant articles published between January 2017 and January 2024 were identified; from the findings of these studies, the effectiveness of nutrition education in Saudi Arabia was assessed.

**Results:**

The studies reviewed included children, adolescents, and adults in various regions of Saudi Arabia, with study durations ranging from 2 months to 2 years. In addition to changes in BMI and eating behaviors, four interventions showed significant improvement of physical activity, sedentary behavior, and body image satisfaction, as well as improvements in nutritional knowledge and eating habits. Despite a lack of statistically significant outcomes, five studies documented positive changes and beneficial impacts. Another study reported improved attitudes and behaviors toward healthy diets among teenagers, as well as improvements in nutritional understanding and dietary practices among school staff and students. However, one study revealed that its nutritional intervention was not adequate in providing education about physical exercise and another found no discernible changes in adolescents’ anthropometric measurements, physical activity, or harmful behaviors after an education intervention.

**Conclusion:**

Nutrition education interventions especially school based done in Saudi Arabia, had significantly improved nutritional knowledge, physical activity, body image satisfaction and BMI.

## Introduction

Dietary and lifestyle choices have changed since 1980, with a noticeable rise in eating out and at restaurants, leading to bigger portion sizes (1). Energy-dense and nutrient-poor diets dominated by fats, sweets, and processed foods have replaced diets rich in whole foods such as pulses and whole grains, which are low in refined oils and sugars (2, 3).

Four of the top 10 causes of death, including obesity, several types of cancer, and coronary heart disease, are caused by current eating trends (4). Among the leading causes of premature deaths worldwide are cardiovascular illnesses, type 2 diabetes mellitus, and various malignancies. Each year, 17.9 million people die from cardiovascular diseases, followed by 9.3 million from cancer, and 2.0 million from diabetes (5). Poor-quality nutrition and improper eating habits are major contributors to many chronic disorders (6). Diet-related risk factors were responsible for 7.9 million deaths and 187.7 million disability-adjusted life years (DALYs) lost in 2019 (7). Because of facts such as these, nutritional issues have received more attention in the last 10 years.

Children now consume more energy-dense foods, fewer fruits and vegetables, and excessive quantities of low-nutritional-value foods, according to recent studies (8–10). These poor eating habits are important indicators of metabolic problems in later life (11).

Inadequate nutritional awareness may be associated with unhealthy eating behaviors and poor-quality diets (12). Individuals’ self-perceptions of the value of balanced meals and their levels of nutritional awareness are known to influence their dietary choices and nutritional intake (13). Diet quality is directly impacted by nutritional awareness, which is also correlated with socioeconomic characteristics, specifically income and education, which impact the association between nutritional awareness and diet quality (14).

Maintaining a healthy or suitable body weight is facilitated by the establishment of healthy eating habits, which are mostly dependent on nutritional knowledge (15). Eating habits and nutritional knowledge are clearly linked, ensuring that vital nutrients are consumed throughout the life cycle (16). Nutrition education, whether formal or informal, can enhance understanding and positively influence food consumption (16).

Effective nutrition education can help decrease and control non-communicable diseases, including obesity-related diseases and disorders (17). For instance, nutrition education can promote awareness of carbohydrates and appropriate levels of daily carbohydrate intake, and thus help in the management of diabetes (18). In addition, teaching patients about the Dietary Approaches to Stop Hypertension (DASH) diet can help in the management of hypertension. Furthermore, depression, ovarian and breast cancer, obesity, diabetes, and asthma have all been found to be alleviated or prevented by adoption of a Mediterranean diet (19).

Researchers have found that nutrition education motivates children to participate in activities and promotes a general understanding of nutrition. Academic studies have also shown that people are becoming more conscious of the importance of eating a healthy diet, and that such awareness leads to changed eating habits (20). Poor eating habits also directly contribute to future non-communicable diseases. Education on nutrition is essential if individuals are to choose a healthy, balanced diet. The burden of non-communicable diseases may be lessened with the help of such information (21).

To combat childhood obesity, a number of national and international measures have been put into place. For instance, the Commission on Ending Childhood Obesity of the World Health Organization has made six key recommendations, including encouraging the consumption of nutritious foods, encouraging physical activity, promoting healthy eating habits, promoting preconception and pregnancy care, promoting physical activity and diet in early childhood, and promoting school-age children’s health (22). However, because these initiatives are focused on the creation of policies that call for a multi-sectoral approach, their implementation has been sluggish and uneven (23).

Morbidity is now a bigger issue than mortality in the Kingdom of Saudi Arabia, which has practically finished its epidemiological shift (24, 25). Noncommunicable diseases (NCDs), including diabetes and cardiovascular disease, account for 73% of all deaths in Saudi Arabia and are the cause of an increasing medical and economic burden (24–26).

With a significant increase in the use of ultra-processed foods and beverages, Saudi Arabia is experiencing a nutritional shift that is adversely altering dietary patterns and increasing diseases associated with poor nutrition. Furthermore, the deleterious impacts of growing urbanization on dietary habits and lifestyles are speeding up these changes (27, 28).

Concerns over diet quality (DQ) were highlighted by studies conducted in Saudi Arabia that showed bad eating habits, such as a high intake of fats, salt, and sugar and a low intake of fruits, vegetables, dairy products, nuts, and fish (29, 30). A prior study conducted in Saudi Arabia found that poor DQ was highly prevalent and suggested nutrition education that focused on DQ, sustainable nutrition, and eating habits (31). According to a different study, the Saudi population has bad eating habits, failing to consume the necessary amount of healthy food, consuming more fast food and fatty foods, and having poor breakfast and snacking habits (28).

Adult male obesity rates in Gulf nations like Saudi Arabia are over 30%, while adult female obesity rates are higher still, at about 40% (32). NCDs now account for 73% of all deaths in Saudi Arabia, a country which is now undergoing a major shift in its national health profile (33). In the Kingdom of Saudi Arabia (KSA), poor diet was found to be responsible for 17.4% of adult DALYs and 25.6% of adult fatalities in 2017 (34).

The Saudi Arabian Ministry of Health (MOH) has launched four nationwide programs to control the NCDs epidemic throughout the last 20 years. The first was in 2001 and was the first step in addressing NCDs. Through a number of resolutions, a specialist committee was tasked with creating and upgrading the program (35). The Saudi Arabian MOH collaborated with WHO to create the second and third measures to address the rising incidence of NCDs, which were implemented as Country Cooperation Strategies (2006–2011) and (2012–2016) (36). These strategies suggested that the Saudi healthcare system give the idea of health promotion—which includes leading a healthy lifestyle and preventing and controlling noncommunicable diseases—priority. The strategies also called for the creation of an integrated health education and research program, which should be appropriate given the circumstances in Saudi Arabia (33).

The National Executive Plan for NCDs (2014–2025), which comprises a thorough national strategy to combat NCDs, is the fourth effort. The goal of this approach is to manage and stop NCDs from getting worse (37). This strategy is in line with the Gulf Cooperation Council’s (GCC) NCD resolutions (38) and the WHO Global NCD Action Plan 2013–2020, which aims to meet the WHO’s targets of a 25% reduction in premature mortality from NCDs among individuals aged 30–70 years over a 15-year period (39). Initiating national NCD prevention strategies and supporting multisectoral activities in implementing preventive interventions known as “best buy” interventions are two of the guidelines provided by the global action plan to Member States. By focusing on common risk factors, the most effective interventions are available to lessen the burden of NCDs at the community level. These include encouraging physical activity, increasing costs on alcohol and tobacco, enforcing laws against alcohol and tobacco advertising, removing trans-fat from food supply chains, cutting back on salt intake, and early detection and treatment of noncommunicable diseases. According to WHO, these interventions were appropriate for use in health systems, cost-effective, and supported by evidence (39).

In accordance with WHO goals to enhance population health, strengthen the healthcare system, and better manage noncommunicable diseases, the Saudi Arabian government has put in place a variety of policy initiatives (40). In 2013, the Saudi MOH Executive Council passed a resolution establishing the Obesity Control Program. The Assistant Agency for Primary Healthcare, a division of the MOH’s Agency for Public Health, is home to the Genetic and Chronic Diseases Control General Department, which oversees this initiative. The program is founded on a clear vision and mission to end obesity, provide the best protection, and provide integrated healthcare services to individuals with any of the three levels of obesity in order to improve the health of Saudi Arabia’s population across all age groups (41).

The Saudi Guidelines for the Prevention and Management of Obesity were created at the national level to offer evidence-based strategies for addressing childhood and adult obesity (42). An intervention designed to lower the body weights of adolescents by 5% or more was reported in a recent Saudi study by Al-Daghri et al. (43); this intervention was carried out in Riyadh, and involved a sample of 363 adolescents aged between 12 and 18 years. Classroom-based instructional sessions were used to promote physical activity and provide teaching materials about healthy eating. When adherent and non-adherent groups were compared after 12 months, substantial decreases in BMI were recorded in the adherent group (43).

The purpose of this review was to examine systematically the impact of nutrition education interventions in Saudi Arabia by assessing changes in nutritional knowledge and habits by widening the search through Saudi studies published between 2017 and 2024. This time frame was selected to guarantee that the assessment takes into account the most current and pertinent data about nutrition education in Saudi Arabia. Because public health initiatives and nutrition-related programs have significantly increased in Saudi Arabia since the introduction of Saudi Vision 2030, and it enables the inclusion of recent studies that reflect current practices, policies, and population health behaviors.

## Methods

### Literature search strategy

A comprehensive literature search was undertaken, utilizing databases such as PubMed, Scopus, Google Scholar, Web of Science, and Cochrane, to identify relevant articles published between January 2017 and January 2024. In addition, papers published in regional journals that might not be indexed in global databases were captured by incorporating regional databases like Saudi Digital Library, Arab World Research Source. Thus, a more thorough representation of nutrition education initiatives in Saudi Arabia was guaranteed by this all-encompassing strategy. A thorough search approach was used, combining important phrases associated with public health, nutrition education, and Saudi Arabia utilizing the Boolean operators “AND” and “OR.” The final search term that was used was: AND (“public health” OR “health promotion” OR “community health” OR “health behavior” OR “health outcomes”) AND (“Saudi Arabia” OR “Kingdom of Saudi Arabia” OR “KSA”) AND (“nutrition education” OR “nutrition intervention” OR “health education” OR “dietary education”). The studies included were randomized controlled trials (RCTs), quasi-experimental or observational studies and the population focus were students and adults. A thorough examination of collected references was carried out in search of potentially related literature. The titles and abstracts of every article that was retrieved were separately examined by the reviewer who was trained in public health, nutrition and epidemiology. After that, the whole texts of studies that might be eligible were examined in light of the established inclusion and exclusion criteria. The protocol for this review adhered to the Preferred Reporting Items for Systematic Reviews (PRISMA) standards.

### Inclusion and exclusion criteria

Studies were eligible if they included nutrition education in any population group (children, adolescents, or adults) in Saudi Arabia. Given its critical role in promoting adequate early-life nutrition, they included treatments targeting mother and newborn nutrition, such as breastfeeding instruction. The PICOS eligibility criteria (Population, Intervention, Comparator, Outcomes, Study design) criteria were as follows:

- Population: Children, adolescents and adults residing in Saudi Arabia.
- Intervention: Nutrition education interventions delivered in any format focusing on nutritional habits.
- Comparator: Usual care, no intervention, or other types of interventions.
- Outcomes: Any reported outcomes related to nutrition knowledge, dietary behavior, or health status as reduction in BMI, change in fruits or vegetables intake, etc.
- Study design: Quasi-experimental studies, Cross-sectional studies, randomized controlled trial and pre-post intervention studies.

The methodological quality of the included studies was assessed using a critical appraisal checklist. The reviewer independently evaluated each paper, and discrepancies were settled by discussion. Domains like selection bias, comparability, outcome measurement, were evaluated by the tool and any disagreements were resolved through discussion or consultation with another re-viewer. Studies were excluded if they did not involve a dietary intervention in Saudi Arabia or did not focus on the outcomes and impact of these interventions. Studies published in a language other than English were also excluded.

### Reporting

**Figure 1:**
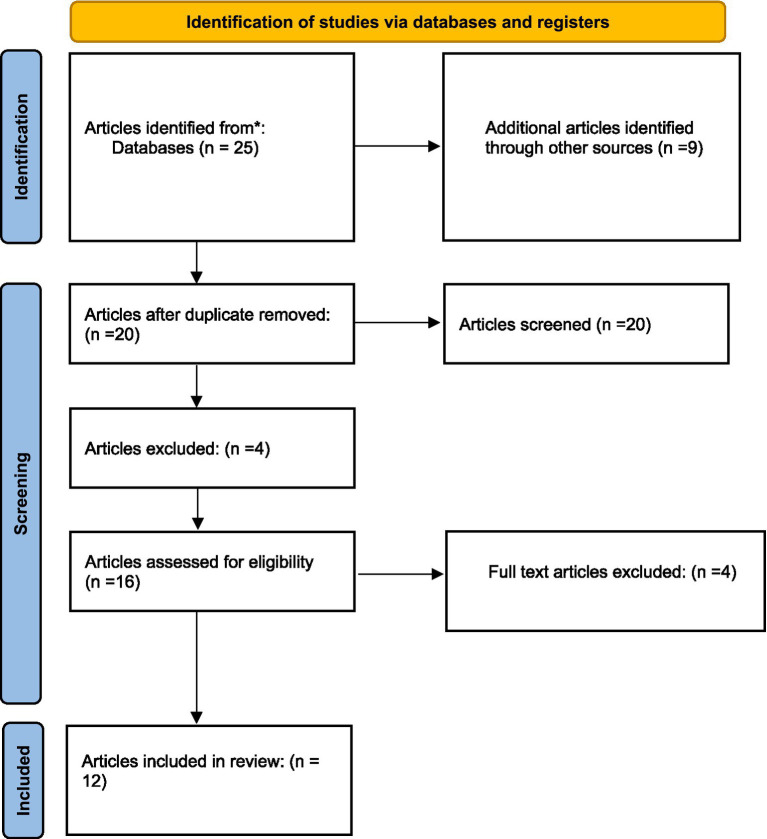
PRISMA flow diagram for the inclusion and exclusion criteria of articles.

The PRISMA guidelines were followed and illustrated in (Figure 1) that was reproduced from an open access source under the Creative Commons Attribution License (CC BY 4.0) (44). Critical appraisal was conducted using Critical Appraisal Skills Program (CASP) tools.

### Data extraction and analysis

The selected studies were thoroughly evaluated to determine their quality and relevance to our study objectives. To gather relevant information from the selected research, a standardized data-extraction form was developed. The following data were extracted, summarized, and presented descriptively: study characteristics, study design, participant information, intervention, findings, and conclusion.

### Assessment of risk of bias in studies

The methodological quality of the 12 chosen studies was assessed, and studies with a high risk of bias were excluded. The risk of bias was measured using pre-specified questions for each research design (Table 1) (45).

**Table 1:** Risk of bias assessment by critical appraisal skills program (CASP) tool.

No.	Study	Clear aims	Methodology	Recruitment	Data collection	Ethical issues	Rigor of analysis	Findings	Value	Overall risk of bias
1	Alahmed et al. (46)	Yes	Yes	Yes	Yes	Yes	Yes	Yes	Yes	Low
2	Banany et al. (47)	Yes	Yes	Yes	Yes	Not clear	Yes	Yes	Yes	Moderate
3	Gosadi (48)	Yes	Yes	Yes	Yes	Yes	Some concerns	Yes	Yes	Moderate
4	Zaghamir and Ibrahim (15)	Yes	Yes	Yes	Yes	Yes	Yes	Yes	Yes	Low
5	Ahmad and Abu Saad (49)	Yes	Yes	Yes	Yes	Not reported	Yes	Yes	Yes	Moderate
6	Almughamisi et al. (50)	Yes	Yes	Yes	Yes	Yes	Yes	Yes	Yes	Low
7	Alzaben et al. (51)	Yes	Yes	Some concerns	Yes	Yes	Yes	Yes	Yes	Moderate
8	Mumena et al. (52)	Yes	Yes	Yes	Yes	Yes	Yes	Yes	Yes	Low
9	Elfaki et al. (53)	Yes	Yes	Yes	Yes	Not clear	Yes	Yes	Yes	Moderate
10	Fetohy et al. (54)	Yes	Yes	Yes	Yes	Yes	Yes	Yes	Yes	Low
11	Kutbi et al. (55)	Yes	Yes	Some concerns	Yes	Not clear	Yes	Yes	Yes	Moderate
12	Alsubaie (56)	Yes	Yes	Some concerns	Yes	Not reported	Yes	Yes	Yes	Moderate

## Results

This systematic review included 12 articles (15, 46–56) of these, six focused on adolescents (47–49, 53–55), two covered both adults and adolescents (50, 51), and three involved adults (41, 42, 48), while only one study included children (57). Geographically, the selected articles covered various regions of Saudi Arabia, with two studies from Jeddah (50, 55), two from Riyadh (51, 54), and two from Jazan (48, 53). Additionally, one article was sourced from each of the following cities: Arar (49), Alkharj (45), Makkah (47), Dammam (46), Madinah (52), and Al-Baha (56).

The study populations varied significantly, ranging from 105 (54) to 38,026 (47) participants in articles focused on adolescents; 34 (50) to 3555 (51) participants in studies including both adults and adolescents, and 46 (52) to 290 (46) participants in studies with adults. In addition, one study examined 725 children (56). Eight interventions were school-based (47–50, 52–54, 56).

School-based interventions (*n* = 8 studies): Most interventions (4, 47–50, 53, 55, 56) were school-based. Four of these reported strong and statistically significant positive outcomes. For instance, Gosadi et al. (48) demonstrated improvements in physical activity and dietary choices; Ahmad and Abu Saad (49) showed sustained improvements in body image satisfaction and physical activity; Almughamisi et al. (50) observed better access to healthy canteen food; and Elfaki et al. (53) reported reduced obesity prevalence alongside improved dietary habits and physical activity.

Other school-based studies indicated positive trends, though without statistical significance. These included increased nutritional knowledge (51), and enhanced dietary practices (54). However, two studies found limited or no effect on anthropometric measures, dietary behavior, or BMI (55, 56).

Community-based interventions (*n* = 4 studies): Four studies (45, 46, 51, 52) were implemented in community or clinical settings. The outcomes were more variable: one study (46) reported nearly threefold improvements in breastfeeding rates with intervention; another (51) found improvements in nutritional knowledge and attitudes. Conversely, one community-based program (52) assessing short-term effects showed limited impact, and another reported suboptimal delivery of physical activity education compared with dietary education (48). Overall, outcomes were heterogeneous, reflecting differences in study populations, intervention content, duration, and settings. School-based programs generally showed more consistent positive effects, while community-based interventions had mixed results, often limited by short follow-up or narrow outcome measures.

The durations of the studies ranged from 2 months to 2 years. Two articles evaluated short-term effects (under 3 months) (52, 55); three articles investigated medium-term intervention effects (3 to 6 months); and one article assessed long-term effects (2 years) (47).

Only one study included a follow-up after 3 months (50). Reviewed studies included participants of both biological sexes, although most individual works targeted either female or male adolescents, due to gender-segregated schooling; only one study of adolescents involved both genders (57).

The primary findings from most of the reviewed studies (Table 2 and Table 3) indicated that four interventions resulted in strong positive and significant effects of the educational interventions. The first was the Gosadi et al. (48) study, where nutritional education had influenced physical activity and eating choices. The second was Ahmad and Abu Saad (49) study, where immediate and persisted follow-up increases in body image satisfaction, sedentary behavior, and physical activity was observed. The third was the study done by Almughamisi et al. (50), which showed improved access to healthy foods in the canteen after intervention. The fourth was the study done by Elfaki et al. (53), where a significant increase in snacks consumption and PA, and decrease in both fast foods consumption and prevalence of obesity was observed after intervention.

**Table 2:** Authors, study designs, locations, and study objectives of included studies.

	Study	Study design	Location in KSA	Sample	Study objective
1	Alahmed et al. (46)	Quasi-experimental study	Dammam	Women (*n* = 290) aged 18 or above	Evaluate the effectiveness of using a woman-centered Web-Based Breastfeeding Educational Resource (WEBBER) in increasing the rate of exclusive breastfeeding at 1 month after birth.
2	Banany et al. (47)	Analysis of pre–post study data	Makkah	38,026 adolescents, both genders (aged 13–18 years)	Analyze the impact of the Rashaka initiative on the body mass index (BMI) of students during the 2016–2017 and 2018–2019 academic years.
3	Gosadi (48)	Cross-sectional study	Jazan	501 adolescent males	Assess the role that schools play in educating and supporting teenagers with regard to managing their weight.
4	Zaghamir and Ibrahim (15)	Quasi-experimental research design with pre–post phases	Al-Kharj	250 nursing students, both genders (aged 18–25 years)	Assess the effectiveness of an intervention on nursing students’ eating habits and nutrition knowledge and practices.
5	Ahmad and Abu Saad (49)	Cluster-randomized controlled trial	Arar	138 intermediate-school girls (adolescent females)	Analyze the results of physical-activity, nutrition, and body-image-perception interventions.
6	Almughamisi et al. (50)	Concept mapping, a mixed-method approach	Jeddah	19 adults and 15 students at intermediate schools for girls (aged 13–15 years)	Examine the opinions of parents, Ministry of Education (MoE) representatives, school personnel, and students regarding significant and workable intervention options for prevention of obesity in adolescent girls.
7	Alzaben et al. (51)	Pre-test–post-test non-randomized experimental study	Riyadh	3,555 female staff and students	Evaluate the impact of nutrition education and intervention initiatives on staff and student dietary practices and nutrition knowledge.
8	Mumena et al. (52)	Quasi-experimental pre-test–post-test control group study	Madinah	46 females (aged 19–24 years)	Assess the success of a nutrition education program aimed at reducing the consumption of added sugar by female undergraduate students.
9	Elfaki et al. (53)	Pre–post quasi-experimental study	Jazan	565 females (aged 12–15 years)	Examine the efficacy of a healthy lifestyle intervention that incorporates eating behaviors (EH) and physical activity (PA).
10	Fetohy et al. (54)	Intervention study	Riyadh	105 females (aged 11–18 years)	Evaluate teenage girls’ knowledge, attitudes, and behaviors about healthy eating practices before and after a nutrition education program.
11	Kutbi et al. (55)	Randomized control trial	Jeddah	148 males (aged 10–15 years)	Examine changes in anthropometric measurements, sedentary lifestyle choices, good food consumption, and physical activity levels
12	Alsubaie (56)	Multistage-stratified cross-sectional survey	Al-Baha city	725 children (aged 7–12 years)	Examine how school-provided nutrition instruction affects children’s eating habits and body weight

**Table 3:** Key findings and conclusions of included studies.

	Study	Results	Conclusion
1	Alahmed et al. (46)	Women who received the WEBBER intervention had an almost threefold higher rate of exclusive breastfeeding 1 month after giving birth than women who did not receive the intervention.	In a hospital setting where infant formula is routinely given, the Web-Based Breastfeeding Educational Resource (WEBBER) intervention was successful in increasing breastfeeding initiation and exclusive breastfeeding rates, both during the hospital stay and at 1 month after delivery.
2	Banany et al. (47)	After 2 years of implementation, BMI decreased dramatically at all schools. The only significant predictors of BMI change, according to regression modeling, were school gender and educational attainment. Compared with boys and students in secondary schools, girls and students in intermediate schools experienced larger BMI reductions.	The students’ BMIs may have decreased as a result of the Rashaka initiative’s implementation; disparities observed with respect to gender and educational level call for additional research into the program’s execution to ascertain how it might be enhanced to better support boys and secondary-school students in lowering their BMI.
3	Gosadi (48)	The most frequently mentioned sources of weight-loss support were the students’ families and community facilities; the role played by education on physical activity was smaller than that of education regarding eating choices.	Some adolescents expressed dissatisfaction with their body weight, helping to explain why some people who are classified as underweight or normal-weight have tried to lose weight in the last 3 years. Schools in the southern region of Saudi Arabia are not doing a good enough job of encouraging students to engage in physical activity and healthy eating habits. Schools’ efforts to encourage the adoption of healthy lifestyles must be strengthened.
4	Zaghamir and Ibrahim (15)	When comparing the post-test with the pre-test, knowledge and practice scores significantly increased.	After the program was put into place, nursing students’ scores on knowledge and practical application of nutrition and eating habits significantly improved. It is crucial that all medical faculties in Saudi Arabia implement organized nutrition education programs and encourage a healthy diet.
5	Ahmad and Abu Saad (49)	The intervention group experienced immediate and notable increases in body image satisfaction, sedentary behavior, and physical activity; and these changes persisted at follow-up.	In females aged 13 and 14 years, the educational intervention showed notable improvements in body image satisfaction (BIS), sedentary behaviors (SBs), and physical activity (PA) at the follow-up and post-intervention time points.
6	Almughamisi et al. (50)	While adults prioritized school-based education and food provision, there was a lack of communication regarding students’ emphasis on access, both to healthy foods and to physical activity in schools and the wider environment (such as retail settings). Following additional consultations, both stakeholder groups decided to improve access to healthy foods in the canteen.	A canteen-based intervention was deemed both important and practical by students, school personnel, and Ministry of Education (MoE) workers, in order to improve food habits and, thus, assist in preventing obesity among adolescent girls.
7	Alzaben et al. (51)	Practice scores did not significantly alter following intervention, but knowledge ratings were noticeably higher than they were prior to intervention.	Although students’ nutritional knowledge increased as a result of the nutrition awareness program, their eating habits did not significantly change. Peer nutrition education programs, nutrition support interventions, awareness campaigns, and participant follow-ups would all benefit from a longer duration, in order to enhance staff and student food habits.
8	Mumena et al. (52)	The intervention group consumed substantially less added sugar at follow-up than they did at baseline. On average, students in the intervention group consumed much less added sugar than those in the control group.	More than half of the pupils in the intervention group consumed less added sugar as a result of the nutrition education intervention; the community might effectively implement this strategy to reduce added sugar intake.
9	Elfaki et al. (53)	After the intervention, the EH and PA of the schoolchildren in the intervention groups significantly improved, they consumed snacks more frequently, consumed fewer fast foods, and the prevalence of obesity decreased.	Implementation of healthy living programs that are incorporated into Jizan school health programs is necessary for the nutrition intervention to be effective.
10	Fetohy et al. (54)	Students’ attitudes and behaviors about healthy eating habits significantly improved following the intervention. Following the intervention, the knowledge score did not increase. The overall barrier scores dramatically decreased, and there was a notable improvement in the consumption of fresh food, whole grains, and in eating breakfast every day. There were considerable improvements in checking food expiration dates and avoidance of fatty meals.	As a result of this nutrition education program, adolescents’ attitudes and behaviors with regard to a healthy diet changed. Food-related behaviors that saw substantial changes included eating breakfast, fresh food, and whole grains, as well as avoiding fatty meals and checking product expiration dates.
11	Kutbi et al. (55)	The number of students in the intervention group who consumed fast food and French fries decreased, but the percentage of children who consumed fruits and vegetables daily and met the necessary daily activity requirement increased.	The provided intervention had no discernible effect on the adolescents’ anthropometric measurements, levels of physical activity, or unhealthy behaviors.
12	Alsubaie (56)	Regarding food habits and BMI, no differences were found between the groups that did report receiving instruction on healthy nutrition and those that did not.	The school-based nutrition education program was found to be insufficient, ineffectual, and inadequate.

Five studies reported improvements and positive effects, although without statistically significant results; for example, the rate of exclusive breastfeeding was nearly three times higher among women who participated in the WEBBER intervention (46). Another study noted enhancements in nutritional knowledge and dietary practices among school students and staff, along with better attitudes and behaviors related to healthy diets among adolescents (51). However, one study found that the provision of education concerning physical activity was suboptimal, compared with education about eating habits (48). Additionally, one study found that completing a nutrition education program had no significant effects on anthropometric measures, physical activity, or unhealthy behaviors in adolescents (55). Lastly, no differences were observed in dietary behaviors and BMI between groups that reported being taught about healthy nutrition and those that did not (56).

## Discussion

In this review, we aimed to assess the impact of nutrition education in Saudi Arabia, as reported in studies published between 2017 and 2024.

Eight of the studies included in this review involved adolescents (47–51, 53–55). Obesity in children and adolescents has been identified as a public health problem in both developed and developing countries (57). The Gulf Cooperation Council (GCC) nations—Saudi Arabia, the United Arab Emirates, Kuwait, Bahrain, Oman, and Qatar—have seen significant sociocultural and lifestyle changes in recent decades. An increased prevalence of high-calorie diets, sedentary lifestyles, and physical inactivity has led to a sharp rise in childhood obesity (58). In one 2022 report, 25.7% of school-age children in Saudi Arabia were classified as overweight or obese (59).

Among the 12 articles reviewed, eight studies described interventions carried out in schools (47–50, 53–56). Given that students spend a large portion of their time in schools, and because schools offer meal programs and physical education classes, among other environmental elements, schools can be seen as ideal places to undertake weight-related interventions (60, 61).

Five of the eight school-based studies in this review demonstrated that nutritional intervention positively improved the participants’ behaviors (47, 49, 50, 53, 54). In a recent study by Raut et al., school-age teenagers’ nutritional knowledge and attitudes were found to be improved by a health education intervention (62). In addition, a systematic analysis of 12 studies published between 2020 and 2023 revealed that school-based nutrition education interventions based on behavior change theories and models had a favorable impact on the eating habits of teenagers (63).

The promotion of healthy food intake, the promotion of physical activity, preconception and pregnancy care, early childhood diet and physical activity, and school-age children’s health are among the six primary recommendations made by the World Health Organization’s Commission on Ending Childhood Obesity (64). However, in three trials (48, 55, 56), interventions were found to be unsuccessful. In the study conducted by Gosadi et al. (48), it was found that schools in the southern region of Saudi Arabia were not doing enough to encourage children to be physically active and eat healthily. In the work of Kutbi et al. (55), it was discovered that adolescents’ anthropometric measurements, physical activity, and unhealthy behaviors were not significantly impacted by the given intervention. In the third study, it was found that a school-based nutrition education program was both inadequate and ineffective (56).

A study carried out in Australia also revealed a limited impact of school-based interventions; in this work, it was found that physical-activity, diet, or combination interventions had no effect on children’s weight or quality of life (65). Similar findings were made by researchers in China, who found that school-based interventions had only a minimal impact on childhood obesity (66). In addition, Habib-Mourad et al. assessed a nutritional intervention in Lebanon. In this work, it was stressed that neither the physical attributes of the school nor the availability of nutritious food options could be altered; had this not been the case, the diets of students might have been improved more significantly (67).

The interventions included in the studied articles showed that nutritional education had influenced physical activity and sedentary behavior, eaten choices as healthy foods consumption and decreased fast food, and body image satisfaction.

The study by Faiz et al., demonstrated the efficacy of nutritional education by seeing significant weight variations between the intervention group and the control group. With each follow-up over time, the experimental group’s weight, waist circumference, waist-to-hip ratio, and body fat % all significantly decreased in comparison to the controls (68). This result agrees with previous studies which showed that nutrition education has been a promising solution to improve dietary habits (68–70).

The current review included six studies that focused on adolescents (47–49, 53–55). Healthy eating habits are more likely to be followed by adolescents who have a solid understanding of nutrition (71, 72). Thus, it is crucial to teach kids about nutrition and stress the value of eating a well-balanced diet. One possible way to enhance eating habits has been through nutrition education (71, 72).

Numerous researches among adolescents have supported nutrition education as an effective means of disseminating nutrition knowledge (68, 73, 74). A systematic review that focused on the impact of nutritional education interventions on enhancing adolescents’ general health and well-being by encouraging healthier food choices and cultivating positive body image perceptions also disclosed the effect of nutrition education on body image perception (75). Additionally, prior research has shown that nutrition education has a good effect on increasing physical activity (76, 77).

Of the 12 studies included in the present review, only one assessed long-term effects (2 years) (47), while three investigated medium-term intervention effects (3 to 6 months) (15, 49, 51). Short-term effects (under 3 months) were reported in two articles (52, 55). According to Hoelscher et al. (78) interventions to combat childhood obesity are needed in low-income and ethnically diverse settings. A combination of primary and secondary techniques is expected to offer adequate exposure, resulting in considerable reductions in childhood obesity. There have been few studies on the long-term impacts of experiential nutrition education programs in schools. However, in one such study, conducted in 2024, it was found that giving hands-on nutrition instruction to children could have long-term benefits (79). The positive effect of long-term nutrition intervention of dietary behavior was observed in previous studies (80, 81).

In nine out of the 12 studies considered in the present review, improvements in intended outcomes upon program completion were demonstrated. In the study by Al-Daghri et al. that was carried out in Riyadh, Saudi Arabia, involving a sample of 363 adolescents between the ages of 12 and 18, an intervention was created with the goal of reducing the adolescents’ body weight by 5% or more. As part of the intervention, classroom-based instructional sessions were used to promote physical activity and provide teaching materials about healthy eating. After 12 months, when adherent and non-adherent groups were compared, substantial decreases in BMI were recorded in the adherent group (56).

Bashatah noted that focusing on food intake and encouraging physical exercise could improve nutritional knowledge and behaviors in students, thereby demonstrating the efficacy of nutrition education programs (82). In a study conducted in Ghana in 2024, it was found that school-based food and nutrition education interventions improved children’s awareness of nutrition and increased their consumption of fruit, but had no impact on anthropometric indices (83).

In a recent systematic review, it was found that lower caloric intake, increased consumption of fruits and vegetables, and reduced consumption of sugar-sweetened beverages were all promoted by nutrition education initiatives; in addition, improved knowledge, beliefs, and habits related to nutrition were all encouraged (84). Similar findings have also been reported in other studies (70, 85).

In order to properly prevent overweight and obesity in children and adolescents, the Saudi Guidelines for the Prevention and Management of Obesity also suggest creating long-lasting school-based interventions. An inter-sectoral strategy involving families should be taken into account in these interventions. Unfortunately, there is little information on the extent to which Saudi Arabian schools offer school-based interventions to help children and teenagers manage their weight (56).

To support future research and interventions that address the nutritional needs of the Saudi population, this systematic review provides a summary of empirical evidence to improve nutritional knowledge, improve dietary habits, increase physical activity, boost body image satisfaction, address rising obesity rates, increase exclusive breastfeeding rates, and reduce added sugar consumption among adults, adolescents, and children.

## Limitations

The goal of the research presented in this study was to thoroughly describe and assess the body of knowledge regarding nutrition education initiatives carried out in Saudi Arabia. Our results were arranged according to the study’s design, location, sample, goals, and main results. The ability to prove causation is limited because most of the included research were cross-sectional, pre-post, or quasi-experimental in design. Furthermore, the overall quality of the evidence is diminished by the very small number of randomized controlled trials, which therefore limits the findings’ applicability to larger groups and contexts. Additionally, long-term follow-up to evaluate the durability of behavioral changes across time was absent from the majority of interventions as only one study tracked participants for up to 2 years, raising questions about how long-lasting the effects were. Furthermore, the possibility for a total lifestyle transformation was limited since physical activity components were either underemphasized or not included in nutrition instruction. These drawbacks show that more thorough, extensive, and carefully monitored research is required, especially randomized controlled trials with long-term follow-up, to bolster the body of evidence and direct Saudi Arabian nutrition education policy and practice. In addition, future studies should concentrate on creating multimodal therapies that incorporate education on physical activity and nutrition, with extensive follow-up times to assess long-term effects.

## Conclusion

According to this review, nutrition education initiatives in Saudi Arabia have the potential to raise eating habits and increase nutritional awareness among a range of age groups. The evidence is conflicting, though, and variables in study design, intervention duration, delivery strategies, and cultural adaptability are probably what affect effectiveness. Few programs assessed the long-term sustainability of these changes, despite the fact that many reported excellent short-term results. To increase their impact on public health, future programs should concentrate on putting into practice evidence-based, culturally appropriate interventions with integrated lifestyle elements and thorough assessment.

## Data Availability

The original contributions presented in the study are included in the article/supplementary material, further inquiries can be directed to the corresponding author/s.
